# Proteomic analysis of human aqueous humor using multidimensional protein identification technology

**Published:** 2009-12-11

**Authors:** Matthew R. Richardson, Marianne O. Price, Francis W. Price, Jennifer C. Pardo, Juan C. Grandin, Jinsam You, Mu Wang, Mervin C. Yoder

**Affiliations:** 1 Department of Pediatrics, Indiana University School of Medicine, Indianapolis, IN; 2 Cornea Research Foundation of America, Indianapolis, IN; 3 Monarch LifeSciences, Indianapolis, IN

## Abstract

Aqueous humor (AH) supports avascular tissues in the anterior segment of the eye, maintains intraocular pressure, and potentially influences the pathogenesis of ocular diseases. Nevertheless, the AH proteome is still poorly defined despite several previous efforts, which were hindered by interfering high abundance proteins, inadequate animal models, and limited proteomic technologies. To facilitate future investigations into AH function, the AH proteome was extensively characterized using an advanced proteomic approach. Samples from patients undergoing cataract surgery were pooled and depleted of interfering abundant proteins and thereby divided into two fractions: albumin-bound and albumin-depleted. Multidimensional Protein Identification Technology (MudPIT) was utilized for each fraction; this incorporates strong cation exchange chromatography to reduce sample complexity before reversed-phase liquid chromatography and tandem mass spectrometric analysis. Twelve proteins had multi-peptide, high confidence identifications in the albumin-bound fraction and 50 proteins had multi-peptide, high confidence identifications in the albumin-depleted fraction. Gene ontological analyses were performed to determine which cellular components and functions were enriched. Many proteins were previously identified in the AH and for several their potential role in the AH has been investigated; however, the majority of identified proteins were novel and only speculative roles can be suggested. The AH was abundant in anti-oxidant and immunoregulatory proteins as well as anti-angiogenic proteins, which may be involved in maintaining the avascular tissues. This is the first known report to extensively characterize and describe the human AH proteome and lays the foundation for future work regarding its function in homeostatic and pathologic states.

## Introduction

The aqueous humor (AH) is a clear fluid that fills the anterior segment of the eye and bathes the lens, iris, and corneal endothelium [1]. It is secreted by the ciliary body and functions to provide nutrients and remove waste from avascular tissues [2], as well as create the intraocular pressure that maintains the convex shape of the cornea. The AH has antioxidant properties and purported immune response roles during inflammation and infection [3,4]. However, the proteins that are responsible for carrying out these functions are largely unknown. The protein content of the AH has been studied extensively [5-12]; however, due to limitations in technology, an extensive and definitive description of human AH proteins has yet to be provided. Furthermore, proteins in the AH are thought to be involved in development of several eye diseases [13,14], and investigating the AH proteome will facilitate generation of new hypotheses regarding the etiology of such pathologies. Thus our goal in this study was to investigate the AH proteome and determine its protein constituents with high confidence using an advanced proteomic approach.

Previous AH proteomic studies were limited by a number of factors including interfering high abundance proteins, inadequate animal models, and limited proteomic technologies. In 1998 Rohde et al. [5] used a proteomic approach to analyze AH proteins but because of technological limitations, proteins were identified based solely on molecular weight and thus must be considered tentative. In 2005 Funding et al. [15] and in 2008 Duan et al. [16] each used two-dimensional electrophoresis in combination with tandem mass spectrometry to identify seven unique AH proteins with high confidence, and in each study interfering high abundance proteins such as albumin limited the depth of the analysis. Also, in 2007 Stastna et al. [1] used an elaborate combination of proteomic techniques to identify almost a hundred proteins from rabbit AH; however, the rabbit genome is incomplete so tentative cross-species identifications had to be made using a broad mammalian database. Here we employed an albumin/IgG depletion strategy to remove these interfering abundant proteins in human derived AH, thus yielding two fractions: albumin-bound and albumin-depleted. For each fraction, Multidimensional Protein Identification Technology (MudPIT) was utilized, which incorporates two-dimensional liquid chromatography coupled with tandem mass spectrometry (2D-LC-MS/MS). In the first liquid chromatography dimension, peptides are separated by strong cation exchange chromatography thereby reducing sample complexity before separation in the second dimension, standard reversed-phase high performance liquid chromatography. In the final step, data are collected using tandem mass spectrometric analysis. This led to an unprecedented high number of definitive human AH protein identifications.

## Methods

The study subjects were patients who were scheduled to undergo standard cataract surgery at a tertiary referral center, Price Vision Group (Indianapolis, IN). Exclusion criteria were as follows: previous intraocular surgery, history of conjunctivitis or any ocular infection within the previous 3 months, intraocular inflammation, or any eye disease. An independent Institutional review board (IRB) approved the study and all subjects signed a written Informed Consent document. Before undergoing cataract surgery, the patient's eye was anesthetized topically with proparacaine. A stab incision was made in the peripheral cornea, and 0.1 to 0.2 ml of anterior chamber fluid was aspirated using a 30-gauge needle. Aqueous humor samples were stored frozen in liquid nitrogen until analysis. A single surgeon (F.W.P.) collected all the samples. Any sample suspected of being contaminated with blood or iris pigment was discarded. Samples from 12 subjects were analyzed; 50% were from females, mean age was 65±6.3 years, 11 were Caucasian, and 1 was Asian (Table 1).

**Table 1:** Patient Data.

**Age**	**Sex**	**Race**	**Contact lens**	**Surgical history**
63	F	Caucasian	No	Lasik
57	F	Caucasian	No	Anterior lamellar keratoplasty
59	F	Caucasian	No	NA
68	M	Caucasian	No	NA
72	M	Caucasian	No	NA
74	M	Caucasian	No	NA
60	F	Caucasian	No	Lasik
59	M	Asian	Yes	NA
70	F	Caucasian	No	NA
65	M	Caucasian	Yes	Lasik
62	F	Caucasian	Yes	NA
75	M	Caucasian	No	NA

The following sample preparation and mass spectrometric analyses were carried out at Monarch LifeSciences (Indianapolis, IN). All reagents and chemicals were purchased from Sigma-Aldrich (St. Louis, MO) unless indicated otherwise. AH samples were pooled and fractionated into bound and flow-through (depleted) fractions using Sigma-Aldrich ProteoPrep Immunoaffinity Albumin & IgG Depletion Kit. The fractionation was performed via manufacturer’s procedures. Protein concentration for each fraction was determined by the BCA protein concentration assay (Bio-Rad, Hercules, CA). The albumin-depleted fraction contained 116 µg, and the albumin-bound fraction contained 221 µg of protein. Each fraction was denatured with 8 M urea, reduced with 10 mM dithiothreitol (DTT), and alkylated with 55 mM iodoacetamide. Urea concentration was lowered to 1.6 M with 10 mM Ammonium bicarbonate (pH 8). Trypsin (V5280; Promega, Madison, WI) was prepared by adding 100 µl of ice cold 0.1 M ammonium bicarbonate to the trypsin vial to make a 1µg/µl solution, then added in a 1:20 (trypsin:protein) ratio and incubated overnight at 37 °C. Peptides (100 µl) were precipitated with 800 µl of diethylether and 400 µl of methanol to remove salt. Then, each sample was dissolved in 0.1% formic acid, and 40 µg of protein digest was loaded onto SCX column (Zorbax, Bio-SCXII 35 x 0.3 mm, 3.5 µm) at 3 µl/min with an Agilent 1100 high pressure liquid chromatography (HPLC) system (Agilent Technologies, Santa Clara, CA). Peptides were eluted with a 2.5 mM to 250 mM NaCl gradient for 2 hr collecting fractions every 8 min, thus yielding 16 fractions by strong cation exchange chromatography. Each of these 16 fractions for both the albumin-bound and albumin-depleted fractions was dried and resuspended in 40 µl of 0.1% formic acid. Columns were equilibrated with 3% acetonitrile (ACN) and fractions were loaded onto the trapping column at 5 µl/min. Salt was washed away for 15 min with 3% ACN before peptides were injected onto the C18-reversed-phase column. Zorbax C18 (300 Ǻ, 3.5 µm; Agilent Technologies) resin was used for both the trapping column (200 µm×2 cm, 5 µm) and the analytical column (100 µm×10 cm, 5 µm) and were self-packed in house. Peptides were eluted with a linear gradient from 5 to 45% ACN over 120 min at room temperature, at a flow rate of 500 nl/min, and the effluent was electro-sprayed into the LTQ mass spectrometer (Thermo-Fisher Scientific, Waltham, MA) with a nanospray source. The source was operated in positive ion mode with 3.0 kV electrospray potential, a sheath gas flow of 15 arbitrary units, and a capillary temperature of 200 °C. The source lenses were set by maximizing the ion current for the 2+ charge state of angiotensin. Data were collected in the triple play mode. Maximum ion time was 200 msec for the full scan and 500 msec for the zoom scan and the MS/MS scan.

The acquired data were filtered by a proprietary algorithm that was developed by Higgs, et al. [17] and described in detail in a recent publication. Database searches were carried out using both Sequest (Thermo-Fisher Scientific, Waltham, MA) and X!Tandem algorithms and the human protein database from IPI (version 3.48). Parameters were set as follows: a mass tolerance of 2 Da for precursors and 0.7 Da for fragment ions, two missed cleavage sites allowed for trypsin, carbamidomethyl cysteine as fixed modification, and oxidized methionine as optional modification. The q-value represents peptide false identification rate and was calculated by a recently published method [18] which incorporates Sequest and X!Tandem results in addition to a number of other relevant factors such as Δ [M+H]+ and charge state. Protein identifications were assigned to one of four groups as discussed previously [19] with priority 1 identifications (IDs) regarded as high-confidence and requiring ≥2 unique peptides and a q-value ≤0.1. Priority 2 IDs require only a single unique sequence and a q-value ≤0.1. Priority 3 and 4 IDs require multiple or single unique peptides, respectively, and peptide q-values fall in the range of 0.25-0.1. Gene ontological analyses were carried out using Princeton University’s gene ontology (GO) tools available at. The Swiss-Prot accession numbers listed in Table 2 and Table 3 were used as the input values with the “Generic GO Term Finder” tool. The “goa_human” gene association file was used for both “function” and “component” analyses.

**Table 2:** Proteins bound to albumin in the aqueous humor of patients with cataracts identified using LC-MS/MS.

**Swiss-Prot number**	**Protein common name**	**Plasma protein**	**MW (kDa)**	**Distinct sequences**	**Minimum peptide q-value**	**Peptide sequence with minimum q-value**
229528	751423A protein Len,Bence-Jones	NF	24	3	2.58E-03	TVAAPSVFIFPPSBZZLK
P02649	Apolipoprotein E [1]	Yes	36	2	1.50E-07	SELEEQLTPVAEETR
P10909	Clusterin [19]	Yes	52	6	2.93E-08	EILSVDCSTNNPSQAK
P01034	Cystatin C [1,5]	Yes	16	2	2.93E-08	LVGGPMDASVEEEGVR
Q12805	EGF-containing fibulin-like extracellular matrix protein 1	Yes	55	2	2.93E-08	DIDECDIVPDACK
P23142	Fibulin-1 precursor, Isoform B	Yes	77	3	3.21E-08	SQETGDLDVGGLQETDK
P22352	Glutathione peroxidase 3	Yes	26	2	1.60E-07	NSCPPTSELLGTSDR
P05109	Protein S100-A8 [5]	Yes	11	2	2.24E-02	MLTELEKALNSIIDV
P41222	Prostaglandin-H2 D-isomerase	Yes	21	3	2.93E-08	APEAQVSVQPNFQQDK
P02787	Serotransferrin [1]	Yes	77	8	2.93E-08	IECVSAETTEDCIAK
28590	Unnamed protein product	NF	69	2	4.37E-04	KVPEVSTPTLVEVSR
P02774	Vitamin D-binding protein [1,20]	Yes	53	2	1.90E-07	SLGECCDVEDSTTCFNAK

NF, not found in current list of identified plasma proteins from Anderson et al. [21]. Proteins identified by previous AH proteome analyses (Stastna et al. [1], Rohde et al. [5], Funding et al. [15], and Duan et al. [16]) are marked at the end of the protein name by the appropriate reference.

**Table 3:** Proteins in the albumin-depleted aqueous humor fraction of patients with cataracts identified with high confidence using LC-MS/MS.

**Swiss-Prot number**	**Protein common name**	**Plasma protein**	**MW (kDa)**	**Distinct sequences**	**Minimum peptide q-value**	**Peptide sequence with minimum q-value**
229479	740525A lipoprotein Gln I	NF	28	8	3.42E-06	VSFLSALEEYTK
P43652	Afamin	Yes	69	3	2.01E-07	DADPDTFFAK
P02763	Alpha-1-acid glycoprotein 1 [1]	Yes	24	4	0.00E+00	EQLGEFYEALDCLR
P19652	Alpha-1-acid glycoprotein 2	Yes	24	3	1.42E-07	TLMFGSYLDDEK
P01011	Alpha-1-antichymotrypsin, Isoform 1	Yes	48	7	1.85E-10	AVLDVFEEGTEASAATAVK
P01011	Alpha-1-antichymotrypsin, Isoform 2	Yes	48	3	1.44E-08	LYGSEAFATDFQDSAAAK
P01009	Alpha-1-antitrypsin [1,15]	Yes	47	16	0.00E+00	DTEEEDFHVDQVTTVK
P04217	Alpha-1B-glycoprotein	Yes	54	2	7.26E-05	CEGPIPDVTFELLR
P25311	Alpha-2-glycoprotein 1, zinc	Yes	34	4	3.42E-06	AYLEEECPATLR
P02765	Alpha-2-HS-glycoprotein	Yes	39	4	3.93E-06	EHAVEGDCDFQLLK
P01023	Alpha-2-macroglobulin [1]	Yes	163	5	1.06E-07	NEDSLVFVQTDK
Q06481	Amyloid-like protein 2, Isoform 1	NF	87	2	1.65E-05	VPYVAQEIQEEIDELLQEQR
Q7KZ97	Antithrombin III variant [1]	Yes	53	8	4.50E-06	DDLYVSDAFHK
P02647	Apolipoprotein A-I [1,5]	Yes	31	14	6.42E-09	DYVSQFEGSALGK
Q76EI7	Apolipoprotein A-II	Yes	3	3	5.70E-08	EPCVESLVSQYFQTVTDYGK
P06727	Apolipoprotein A-IV [1]	Yes	45	3	4.82E-06	SLAPYAQDTQEK
P05090	Apolipoprotein D [1]	Yes	21	2	3.17E-08	CPNPPVQENFDVNK
P02649	Apolipoprotein E [1]	Yes	36	5	4.38E-07	VQAAVGTSAAPVPSDNH
P02749	Beta-2-glycoprotein 1	Yes	38	4	3.37E-06	CSYTEDAQCIDGTIEVPK
P61769	Beta-2-microglobulin [5]	Yes	14	3	5.63E-04	VNHVTLSQPK
O94985	Calsyntenin 1 isoform 2	NF	110	2	3.07E-08	AASEFESSEGVFLFPELR
P00450	Ceruloplasmin [1]	Yes	122	20	7.16E-08	HYYIGIIETTWDYASDHGEK
P10909	Clusterin [15]	Yes	52	13	0.00E+00	EILSVDCSTNNPSQAK
P01024	Complement C3	Yes	187	14	2.12E-07	SGSDEVQVGQQR
P02462	Collagen alpha-1(IV) chain [1]	Yes	161	2	0.00E+00	GDPGLKGDK
P0C0L4	Complement C4-A [1]	Yes	193	8	1.24E-08	VLSLAQEQVGGSPEK
P00751	Complement factor B, Isoform 1	Yes	86	3	4.68E-06	EAGIPEFYDYDVALIK
P01034	Cystatin C [1,5]	Yes	16	4	7.15E-08	ALDFAVGEYNK
Q9UBP4	Dickkopf-related protein 3	NF	38	7	4.75E-11	SAVEEMEAEEAAAK
Q12805	EGF-containing fibulin-like extracellular matrix protein 1	Yes	55	2	4.05E-05	IQCAAGYEQSEHNVCQDIDECTAGTHNCR
P06396	Gelsolin, Isoform 1	Yes	86	7	5.52E-07	PALPAGTEDTAK
P22352	Glutathione peroxidase 3	Yes	26	4	2.10E-06	NSCPPTSELLGTSDR
P00739	Haptoglobin-related protein, Isoform 1	Yes	39	4	5.42E-06	AVGDKLPECEAVCGKPK
Q8IZI0	Hemoglobin beta chain variant Hb-I Toulouse	NF	12	7	3.31E-08	VNVDEVGGEALGR
P69905	Hemoglobin subunit alpha [5]	Yes	15	5	4.97E-11	VGAHAGEYGAEALER
P68871	Hemoglobin subunit beta [5]	Yes	16	9	3.31E-08	VNVDEVGGEALGR
A0N071	Hemoglobin subunit delta [5]	Yes	16	4	7.12E-06	VLGAFSDGLAHLDNLK
P02790	Hemopexin	Yes	52	14	2.24E-08	GECQAEGVLFFQGDR
P04196	Histidine-rich glycoprotein	Yes	60	3	2.98E-06	DSPVLIDFFEDTER
Q0VAC5	HP protein	NF	38	7	1.10E-06	TEGDGVYTLNNEK
P10745	Interphotoreceptor retinoid-binding protein	NF	135	11	2.03E-07	TAVDLESLASQLTADLQEVSGDHR
Q2XP30	Mutant beta-globin	NF	2	2	4.24E-05	VHLTPEEK
Q9UBM4	Opticin	NF	37	2	1.51E-07	EGDSFEVLPLR
P36955	Pigment epithelium-derived factor	Yes	46	12	0.00E+00	DTDTGALLFIGK
P05155	Plasma protease C1 inhibitor	Yes	55	4	2.98E-06	LEDMEQALSPSVFK
Q9P173	PRO2275	NF	13	5	3.20E-10	VFSNGADLSGVTEEAPLK
P41222	Prostaglandin-H2 D-isomerase	Yes	21	10	0.00E+00	EAQVSVQPNFQQDK
P02787	Serotransferrin [1]	Yes	77	50	2.57E-11	FDEFFSEGCAPGSK
P02766	Transthyretin [1,15,16]	Yes	16	12	2.24E-08	KAADDTWEPFASGK
P68871	Unnamed protein product	NF	16	8	3.31E-08	VNVDEVGGEALGR
P02774	Vitamin D-binding protein [1,16]	Yes	53	7	3.22E-08	VPTADLEDVLPLAEDITNILSK

NF, not found in current list of identified plasma proteins from Anderson et al. [21]. Proteins identified by previous AH proteome analyses (Stastna et al. [1], Rohde et al. [5], Funding et al. [15], and Duan et al. [16]) are marked at the end of the protein name by the appropriate reference.

## Results

A statistical summary provided in Table 4 indicates the number of proteins identified at each priority level from each fraction collected. The twelve albumin-bound AH proteins identified with high confidence (priority 1) are listed in Table 2, and the fifty AH proteins identified with high confidence (priority 1) in the albumin-depleted (flow-through) fraction are listed in Table 3. Eight of the proteins were common to both fractions; therefore, in total we identified 54 unique human AH proteins in this study. Only the proteins meeting priority 1 criteria (multiple unique peptides with a minimum q-value ≤0.1) were chosen to list in this manuscript as most experts agree that protein identities established by less stringent criteria are questionable [20]. Proteins identified at priority levels 2-4 can be found in Appendix 1. The Swiss-Prot protein accession number, protein common name, and molecular weight are provided in Table 2 and Table 3. Identified proteins were cross-referenced with Anderson et al.’s [21] published list of plasma proteome constituents, and their presence in the plasma is listed in the table as present or not found. Over 80% of the proteins identified in each fraction were previously identified as plasma proteins by Anderson et al. [21]. Also listed are the protein’s molecular weight (kDa), the number of unique peptides identified by MS/MS for each protein, each protein’s minimum peptide q-value, and the peptide sequence corresponding to the peptide with the lowest q-value.

**Table 4:** Statistical summary of protein identification results from the LC-MS/MS experiment in both albumin-depleted and albumin-bound groups.

**Protein Priority**	**Peptide ID Confidence**	**Multiple Sequences**	**Albumin-depleted**		**Albumin-bound**	
			**Median Number of Sequences**	**Number of Proteins**	**Median Number of Sequences**	**Number of Proteins**
1	HIGH	YES	5	50	3	12
2	HIGH	NO	1	35	1	92
3	MODERATE	YES	2	2	2	3
4	MODERATE	NO	1	74	1	94

**Figure 1:**
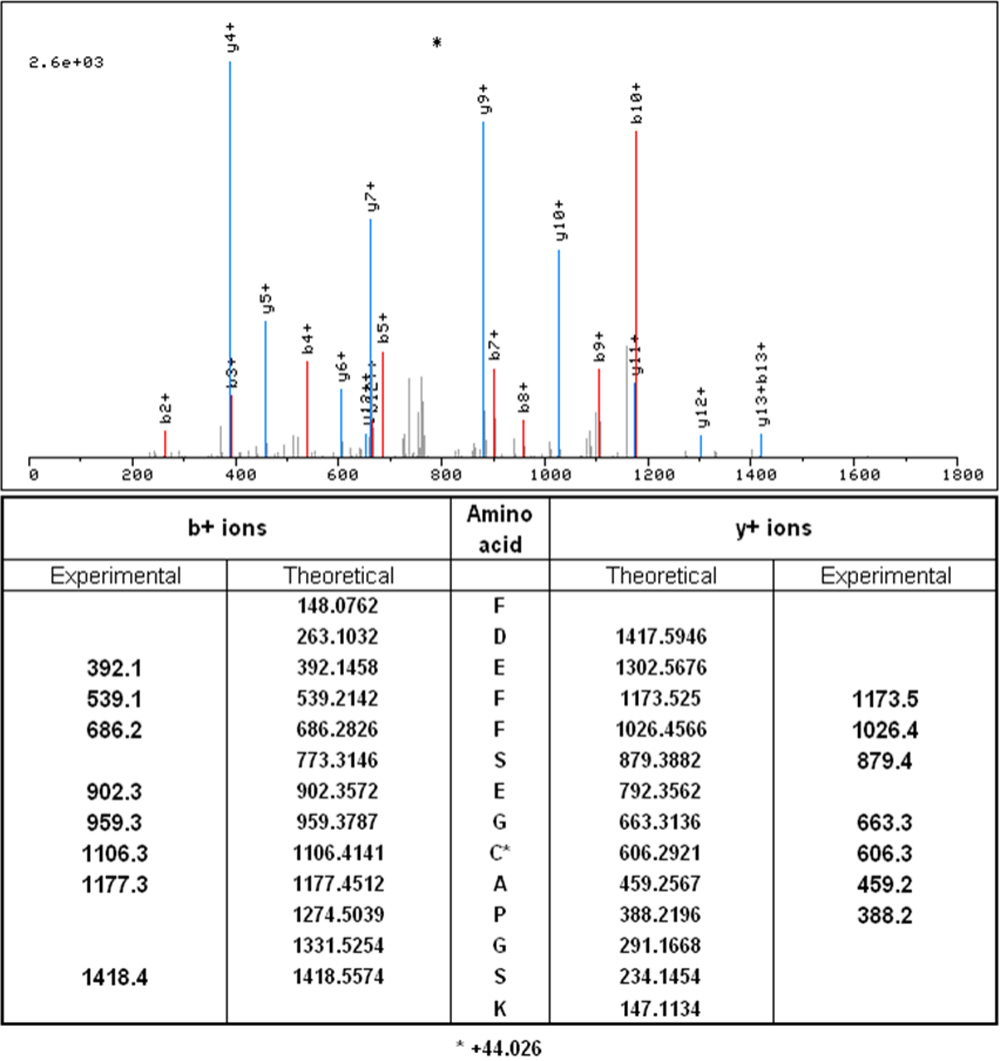
Transferrin peptide tandem mass spectra Tandem mass spectrum of the peptide FDEFFSEGCAPGSK from the protein Transferrin (above). This peptide was identified with high confidence through the matching of the experimental and theoretical m/z values (below) from the peptides’ characteristic b and y ions (delta m/z <0.1).

**Figure 2:**
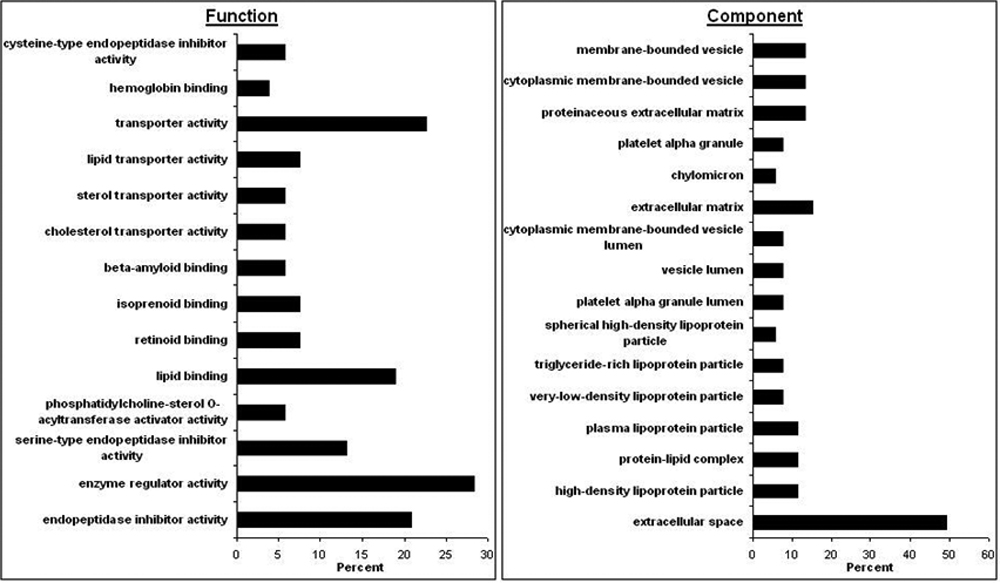
Function and component gene ontological analyses of proteins identified in the aqueous humor. Each bar represents the percent of priority 1 AH proteins (a sum of unique proteins in the albumin-depleted and albumin-bound fractions) that belong to a particular GO term listed on y-axis.

A representative tandem mass spectrum corresponding to a peptide (FDEFFSEGCAPGSK) of the protein transferrin is illustrated in Figure 1. The high mass accuracy measurements of the fragmented peptides’ characteristic b and y ions enabled high confidence matching and contributed to its low q-value. Gene ontological analyses for function and component GO terms were performed, and the results are illustrated in Figure 2. This analysis revealed an enrichment for extracellular space proteins and proteins typically found in lipoprotein particles, as well as for proteins with functions in lipid binding and endopeptidase inhibition.

## Discussion

Rather than focus on differentially expressed proteins in the AH of patients with a particular ocular pathology, we chose to concentrate our efforts on a thorough investigation of the human AH proteome isolated from patients who did not have any significant health problems other than cataracts, which are just part of the normal aging process. We utilized albumin depletion to remove the known interfering high abundant protein and associated proteins and therefore increase lower abundant protein identification. We also incorporated 2D-LC-MS/MS to further fractionate samples and facilitate identification of lower abundant proteins. By doing so, we were able to overcome obstacles of previous AH proteome analyses and provide the highest confidence information to date on the protein composition of healthy human aqueous humor in a population of aged adult subjects.

### Historical context

In 1998 Rohde et al. [5] attempted to tentatively identify lower abundance human AH proteins using membrane preconcentration coupled with capillary electrophoresis and mass spectrometry (mPC-CE-MS). Of the 27 putative protein identifications, the following seven were confirmed in our analysis: beta-2 microglobulin, an S100 calcium binding protein, hemoglobin, Cystatin C, and apolipoprotein A-I as well as albumin and IgG. As the authors astutely noted, protein identification based only on molecular weight (MW) can only be considered tentative until confirmatory evidence, such as amino acid sequence, can be provided as we have done here.

In 2005, Funding et al. [15] used two-dimensional electrophoresis (2-DE) to separate AH proteins and image analysis to determine differentially expressed AH proteins in patients with acute corneal rejection. Then using a combination of immunoblotting, matrix-assisted laser desorption/ionization-time of flight (MALDI-TOF MS), and nanoLC-MS/MS they were able to identify six proteins with high confidence: cytokeratin type II, clusterin (apolipoprotein J), serine protease inhibitor, albumin, alpha-1-antitrypsin, and transthyretin. We did not confirm the presence of cytokeratin, a common protein contaminant in mass spectrometric protein identifications, and we also did not identify serine protease inhibitor specifically, but did identify proteins from the serine protease inhibitor (SERPIN) family such as alpha-1-antichymotrypsin and antithrombin III. The presence of the other four AH proteins were confirmed in our analysis.

In 2007, Stastna et al. [1] employed an elegant combination of 1-DE, 2-DE, and reversed phase liquid chromatography (RPLC) for protein separation and MALDI-TOF MS and LC-MS/MS for AH protein identification. This appears to be the only publication dealing exclusively with the AH proteome, rather than a differential expression analysis, and is thus the most relevant published report of an extensive characterization of the proteome. The major limitation to their approach, however, was the use of a rabbit model for AH analysis. Only 34 of their 98 proteins were identified as rabbit (*O. cuniculus*) proteins with the rest being identified as other various mammalian proteins. This limited the analysis for two reasons: 1) the rabbit is not always useful as a model of human ocular pathology; for example the rabbit corneal endothelium regenerates unlike that in humans [22], and 2) the rabbit genome is currently incomplete, so one must rely upon other mammalian genomes for cross-species identifications, which must be regarded as tentative until the rabbit gene(s) sequence(s) can be confirmed. Thus, the next clear step in AH analysis was an extensive investigation of the human AH proteome. We could only confirm 21 of the 98 rabbit AH protein identifications reported by Stastna et al. [1] in the human AH. These proteins are highlighted in Table 2 and Table 3 with the exception of the following six proteins which were identified outside of priority 1 (priority 2-4, less confident identifications): type II collagen, desmocollin, desmoglein, plasminogen, cathepsin, and retinol-binding protein.

In 2008 Duan et al. [16] used 2-DE to determine differentially expressed protein spots in the AH of patients with myopia. They determined the identities of six differentially expressed protein spots with high confidence using LC-MS/MS. Four of these spots were identified as albumin with the other two being transthyretin and vitamin D-binding protein, which we also identified with high confidence. Thus, albumin clearly interfered with identifying more proteins in the studies of Duan et al. [16] and Funding et al. [15], and this highlighted the importance of the prefractionation step we utilized to deplete interfering high abundance proteins such as albumin and IgG.

### Biological context

Gene ontological analyses performed using the “Generic GO Term Finder” tool for function and component GO terms revealed several interesting findings. The component analysis revealed that almost half of the proteins identified were predicted to be extracellular space proteins and that proteins associated with several different lipoprotein particles were present in the AH, the relevance of which is discussed below. Regarding function, many AH proteins were found to be involved in lipid transport and binding and in endopeptidase inhibition. A manual search of the literature detailed below has revealed a potential role for several of these proteins in a variety of physiological functions of AH, many of which were identified for the first time here.

Lipoproteins in the AH are thought to provide a significant source of lipids for avascular tissues such as the lens in which de novo cholesterol synthesis is considered insufficient to meet even 50% of its total cholesterol need [23,24]. We identified apolipoprotein J (Apo J), also known as clusterin, which has been suggested to enter the AH from corneal endothelium. Apo J is thought to play a role in cell membrane maintenance at tissue-fluid interfaces possibly protecting cells from complement factors in addition to having speculated immunological roles [25], so it is plausible that Apo J may have a similar function in the AH. It is one of seven lipoprotein proteins identified in this study. Interestingly, lipoproteins are also involved in lysophosphatidic acid (LPA) transport. LPA is a phospholipid that is present in the AH and known to modulate a number of cellular responses. Another LPA transport protein identified in this study is Gelsolin, which also has proposed anti-oxidant properties [26]. Alpha-2-glycoprotein, zinc (ZAG), which has proposed immunoregulatory roles, is also proposed to bind fatty acids and regulate lipid metabolism [27]. The interphotoreceptor retinoid-binding protein and transthyretin identified in this study also have roles in fatty acid transport and lipid metabolism, respectively [28,29], yet their specific roles in the aqueous humor remains unclear. Another identified protein, vitamin D-binding protein (DBP), was previously speculated to have a role in the AH and is a multi-functional plasma protein known to transport vitamin D metabolites and bind fatty acids as well as modulate immunological processes [30]. DBP has been found to be associated with certain ocular pathologies [16]. Other lipoproteins are speculated to be involved in ocular pathological processes as well including age-related macular degeneration (AMD) [31].

The roles of several other proteins identified here have already been purported in the AH. For example, though the exact role of Glutathione Peroxidase in the AH is unclear, it is known to protect cells and other proteins from oxidative damage and most likely serves a similar function in the AH [32]. Prostaglandin-H2 D-isomerase is thought to be involved in maintenance of intraocular pressure as well as development and maintenance of the blood-aqueous barrier [33]. Antithrombin III, a protease inhibitor, has been suggested to regulate intraocular pressure by influencing the function of the trabecular meshwork [34]. Alpha-1-antitrypsin, a serine protease inhibitor, has been proposed to protect the cornea from degradation by neutrophil elastase released during inflammation [35]. Cystatin C, a cysteine protease inhibitor, has been found to be secreted by the ciliary body into the AH of rat and mouse eyes at very high concentrations indicating it would bind essentially all cysteine proteases that may leak into the AH [36]. It is reasonable that other protease inhibitors identified here such as alpha-2-macroglobulin (A2M) and alpha-1-antichymotrypsin 1 and 2 could play similarly important roles. Moreover, many proteases and protease inhibitors are thought to be secreted by the ciliary epithelium, indicating that they could have a specific purpose in the AH [37]. Interestingly, A2M can inhibit proteases such as Cathepsin D, which we identified at a priority 2 level (not shown). Cathepsin D is known to degrade Fibulin-1, a potentially important anti-angiogenic protein [38], which we also identified. Thus, A2M may play an important role in protecting the cornea by inhibiting Cathepsin D. Importantly, Cystatin C is also an anti-angiogenic protein [39] as is Fibulin-3, also known as EGF-containing fibulin-like extracellular matrix protein 1 [40].

Anti-angiogenic proteins are key molecules in the AH as there are several tissues that must remain avascular to maintain their function such as the cornea which serves as a “clear window” to the eye. Indeed, it is known that the AH suppresses angiogenesis [41], but the exact proteins responsible remain incompletely understood. We identified a number of proteins with high confidence, some for the first time in the AH, that have also been identified as potent inhibitors of angiogenesis. These include DBP, Beta-2-glycoprotein 1, Collagen alpha-1(IV), and Pigment epithelium-derived factor (PEDF) [42-45]. DBP is modified by membrane-bound β-galactosidase and sialidase of activated B- and T-lymphocytes, and the resultant molecule is known as DBP-maf (macrophages activating factor), which is a key anti-angiogenic protein [46]. PEDF has been shown to inhibit endothelial cell migration in the presence of angiogenic proteins such as VEGF, and inhibits neovascularization of the rat cornea [45]. Its expression in the AH has been associated with lens opacity as well as total anti-oxidant capacity [47]. PEDF levels are inversely correlated with age, and lack of PEDF has been proposed to play a role in the development of cataracts [48]. Other AH proteins identified here that were previously found to be anti-angiogenic elsewhere include Histidine-rich glycoprotein and Opticin [49,50].

### Conclusion

In this study, we employed multiple prefractionation techniques and tandem mass spectrometry to identify lower abundant proteins and extensively characterize the human AH proteome. These multi-peptide, high-confidence, priority 1 proteins, widely regarded as definitive identifications, provide a reliable foundation and should enable the generation of new hypotheses regarding pathologies of the eye as well as normal AH function. Though these IDs should be considered definitive, it is still possible that there were AH proteins not identified with high confidence in this study. Proteins identified at priority levels 2-4 could be considered candidates for future investigation in this regard. The exact number of human AH proteins is unknown, and it is possible that tens if not hundreds of lower abundance proteins exist in the AH and many of these could fall below current detection limits. Nevertheless, based on previous investigations of AH and other body fluids, the AH would not be expected to contain as many proteins as a typical cell lysate. Other areas for future study include determining differences in AH protein levels between patients in different age groups with different ocular pathologies, as well as differences between men and women; aspects that could not be addressed in the current study.

## Appendix 1

To access the data, click or select the words “Appendix 1.” This will initiate the download of a pdf archive that contains the file.
